# Comparative Physicochemical Characterization of Polylactic Acid-Based Dermal Fillers

**DOI:** 10.3390/polym18010084

**Published:** 2025-12-27

**Authors:** Chen-Ying Su, You-Cheng Chang, Pei-Ju Cheng, Hsu-Wei Fang

**Affiliations:** 1 Department of Chemical Engineering and Biotechnology, National Taipei University of Technology, 1, Sec. 3, Zhongxiao E. Rd., Taipei 106, Taiwan; chenying.su@mail.ntut.edu.tw (C.-Y.S.); youchengchang@mail.ntut.edu.tw (Y.-C.C.); aru0857008@gmail.com (P.-J.C.); 2 High-Value Biomaterials Research and Commercialization Center, National Taipei University of Technology, 1, Sec. 3, Zhongxiao E. Rd., Taipei 106, Taiwan; 3 Institute of Biomedical Engineering and Nanomedicine, National Health Research Institutes, No. 35, Keyan Road, Zhunan Town, Miaoli 350, Taiwan; 4 Institute of Oral Tissue Engineering and Biomaterials, National Yang Ming Chiao Tung University, Taipei 112, Taiwan

**Keywords:** polylactic acid, poly(L,-lactic acid), poly(D,L-lactic acid) acid, dermal filler, carboxymethyl cellulose, hyaluronic acid, soft tissue augmentation

## Abstract

Introduction: Polylactic acid can be classified into poly(L-lactic acid) (PLLA) and poly(D,L-lactic acid) (PDLLA) according to their stereoisomeric structures, and both are widely used as dermal fillers for soft tissue augmentation. Although the clinical efficacy of commercially available PLLA- and PDLLA-based fillers has been well established, variations in their physicochemical properties may lead to differences in handling characteristics and clinical performance. A systematic comparison of these properties among different PLA-based fillers remains limited. Materials and Methods: In this study, the physicochemical characteristics of three PDLLA-based fillers (AestheFill, NeoFilera, and Juvelook) and one PLLA-based filler (Sculptra) were evaluated. The analyses included functional group identification, particle morphology and size distribution observation, reconstitution time measurement, osmotic pressure determination, and viscosity assessment. Results: AestheFill and NeoFilera exhibited similar profiles in terms of functional groups, size distribution, osmotic pressure, and viscosity, while NeoFilera and Juvelook showed comparable particle morphologies. Sculptra displayed distinct particle morphology and viscosity, likely attributable to its PLLA composition, yet showed similarities with Juvelook in functional group identification and osmotic pressure. Additionally, the reconstitution times of Sculptra, NeoFilera, and Juvelook were significantly shorter than that of AestheFill. Conclusions: Although the direct correlation between physicochemical characteristics and clinical outcomes warrants further investigation, this comparative analysis provides clinicians with a clearer understanding of the material properties of PLA-based dermal fillers and may assist in the informed selection of appropriate products for individual patients.

## 1. Introduction

Injectable dermal fillers are widely applied in aesthetic medicine, mainly due to their minimally invasive nature and reliable effectiveness in soft tissue augmentation and wrinkle correction. Injectable dermal fillers can be categorized into physical fillers and bio-stimulatory fillers [1]. Physical fillers, primarily hyaluronic acid (HA) and collagen, are key components of the extracellular matrix. By injecting physical fillers, an effect of filling can be observed immediately. However, the biodegradation time of physical fillers is usually short, typically 3 to 12 months for HA fillers and less than 6 months for collagen fillers. Bio-stimulatory fillers include polymethylmethacrylate (PMMA), polylactic acid (PLA), polycaprolactone, and calcium hydroxylapatite. These materials not only cause an initial physical filling effect but also induce inflammatory and minor foreign-body reactions, leading to fibroblast proliferation and collagen synthesis [2,3]. Among all the materials of bio-stimulatory fillers, the degradation period for PMMA is longest, which can be maintained up to 10 years [4]. Therefore, PMMA can also be considered as a permanent filler, while the rest of the bio-stimulatory fillers are semi-permanent fillers with biodegradation periods of approximately 1 to 2 years [5].

Bio-stimulatory injectable dermal fillers have grown in popularity because they promote long-lasting results through stimulating the patient’s own collagen production. Semi-permanent fillers are often preferred over permanent fillers, as they provide durable effects while offering a lower long-term risk profile due to their biodegradability. Currently marketed semi-permanent fillers include AestheFill, Sculptra, NeoFilera, Repart PLA, Gana V, Juvelook, Ellanse, and Radiesse. Ellanse consists of 30% polycaprolactone microspheres suspended in a 70% sodium carboxymethylcellulose (CMC) carrier gel, while Radiesse consists of 30% calcium hydroxylapatite microspheres suspended in a carrier comprising sterile water, glycerin, and CMC [1]. The remaining semi-permanent fillers primarily contain PLA microparticles, with CMC or HA as auxiliary components. Sculptra and Gana V are composed of poly(L-lactic acid) (PLLA), whereas AestheFill, NeoFilera, Repart PLA, and Juvelook are made from poly(D,L-lactic acid) (PDLLA) [6,7,8]. AestheFill, NeoFilera, Repart PLA, Sculptra, and Gana V all use CMC as the carrier, while Juvelook utilizes non-crosslinked HA. All products contain mannitol as an auxiliary component except AestheFill, NeoFilera, and Juvelook.

Once bio-stimulatory injectable dermal fillers are manufactured, some of their physical–chemical characteristics will be analyzed, in addition to an in vivo efficacy analysis of collagen synthesis. Previous research studies have shown that the particle size and surface morphology of PLA microparticles are critical factors in their biological performance. Particle sizes between 20 and 100 μm with smooth surfaces and regular shapes are recommended for safe and effective PLA dermal fillers. When PLA particle size is smaller than 20 μm, giant cell formation and granulomatous inflammation results from the phagocytosis that is induced by macrophages [1]. Conversely, particles larger than 100 μm may be difficult to inject through a needle [6]. Irregular or rough particle surfaces have also been associated with increased clustering of foreign-body giant cells [9]. Consistent with these principles, the particle size of AestheFill, Sculptra, NeoFilera, Repart PLA, Gana V, and Juvelook have all been reported to fall within the optimal 20 to 100 μm range with smooth morphology [6,8,10].

In addition to particle size, the injectability of dermal fillers is influenced by the viscosity of the microsphere suspension after being reconstituted with sterile water. For HA fillers, rheological characteristics are critical because their clinical effect relies heavily on immediate volumization. Injection of highly viscous HA into superficial layers may cause unnatural contours or overfilled appearances [11,12]. Therefore, understanding the rheological properties of HA dermal fillers assists clinicians in selecting an appropriate product. In contrast, the viscosity behavior of PLA dermal fillers has rarely been investigated, as their primary benefits arise from long-term collagen stimulation rather than immediate filling. Since PLA-based dermal fillers are typically administered using small-gauge needles such as 25-gauge to minimize tissue trauma, their viscosity should be examined to assess injectability. Nevertheless, their reconstitution property is clinically important. PLA dermal fillers are supplied as lyophilized or patty-like powder, and it is recommended that sterile water be added and the product be allowed to sit until the microspheres are fully and uniformly dispersed. The reconstitution time is defined as the duration from the addition of sterile water to the lyophilized or patty-like powder until complete dissolution and formation of a homogeneous solution. Adverse events, such as localized ecchymosis, edema, nodules, subcutaneous papules, and granulomas, may potentially result from the inadequate dispersion of PLA microspheres or an insufficient volume of dilution [13,14]. Although many new reconstitution methods have been reported, including a back-and-forth method or warming the vial [15,16], it would be advantageous if PLA fillers could achieve uniform suspension simply by standing for less than 10 min.

Previous studies have thoroughly investigated the physicochemical characteristics of commercially available PLA-based fillers. One study thoroughly compared four PLA-based dermal fillers, including AestheFill, Gana V, Repart PLA, and Sculptra, by examining their composition, crystallinity, particle morphology, and size distribution [6]. In addition, hydrolytic degradation was conducted over a 9-month period to evaluate changes in PLA molecular weight and surface morphology. The results demonstrated that differences in crystallinity and particle morphology significantly influenced degradation behavior. Another study comprehensively compared the morphology of Radiesse and Sculptra, including particle shape, size, aspect ratio, and the proportion of phagocytosable particles (defined as particles smaller than 20 μm) [9]. The authors suggested that these morphological differences may be associated with the distinct inflammatory responses observed clinically. To date, physicochemical analyses of PLA-based dermal fillers have primarily focused on particle size and morphology. However, other properties, such as chemical composition, reconstitution behavior, osmotic pressure, and rheological characteristics, may also play important roles in biological response and clinical handling. Therefore, the physicochemical properties of one PLLA-based and three PDLLA-based bio-stimulatory dermal fillers were systematically evaluated and compared in this study. Functional groups were characterized using a Fourier-transform infrared spectroscope, and particle morphology and size distribution were observed by a field emission scanning electron microscope. In addition, reconstitution time was measured, osmotic pressure was tested using an osmometer, and viscosity were analyzed by a rheometer. By integrating these physicochemical properties, this study provides clinicians with a more comprehensive understanding of the differences among PLA-based fillers, thereby supporting more informed product selection to meet diverse clinical and patient-specific needs.

## 2. Materials and Methods

### 2.1. Sample Preparation

Three poly(D,L-lactic acid) (PDLLA) dermal filler and one poly(L-lactic acid) (PLLA) dermal filler were used in this study. PDLLA dermal fillers included NeoFilera (Diamond Biotechnology Co. Ltd., Taipei, Taiwan), AestheFill^®^ (REGEN Biotech Inc., Seoul, Republic of Korea), and Juvelook (VAIM Inc., Seoul, Republic of Korea). The PLLA dermal filler used here was Sculptra^®^ (Q-Med AB, Uppsala, Sweden). NeoFilera, AestheFill^®^, and Sculptra^®^ also contain sodium carboxymethylcellulose (CMC) as a key component, while Juvelook includes non-crosslinked sodium hyaluronate as a carrier gel [6,7,8].

### 2.2. Fourier-Transform Infrared Spectroscopy Analysis

The functional groups of all dermal filler samples were identified using a Fourier-transform infrared spectroscope (FTIR, Spot-light 200i Sp2 with AutoATR System, Perkin Elmer, Shelton, CT, USA). An amount of 100 mg of sample particles was ground in a mortar and pressed into tablets using a tableting machine for FTIR measurements. The FTIR spectra were recorded over a wavenumber range from 400 to 4000 cm^−1^, and 32 scans were run.

### 2.3. Morphology Observation

The surface morphology of various dermal filler particles was imaged using a field emission scanning electron microscope (SEM) (JSM-7610F, JEOL, Peabody, MA, USA) at 15 kV with a Schottky field emission electron gun at a particular distance. After 200 milligrams (mg) of dermal fillers was dissolved in 8 milliliters (mL) of sterile water for injection (SWFI), the suspended solution was kept at −80 °C for 24 hours (h) and then freeze-dried for another 24 h. The dried samples were fixed to a metal support and coated with gold with a sputter coater (Ion Sputter E101, Hitachi, Tokyo) for imaging. The images were taken at both 100× and 500×. The sizes of various particles from each dermal filler were determined using image J software (version 1.54), and the maximum distance between two points was then measured for irregular Sculptra particles. Lengths of 100 particles were measured to obtain the average particle size, and D_av_ is presented as mean size ± standard deviation.

### 2.4. Reconstitution Test

One milliliter of SWFI was added to 25 mg of dermal filler sample, and the photographs were taken at 0 minute (min). Samples were kept still, and photographs were taken every 5 min. If particles were not dissolved after 15 min, the mixture was vortexed at 2700 revolutions per minute (rpm) until all the particles were evenly suspended, and the reconstitution time was recorded. Three independent samples from each dermal filler were measured.

### 2.5. Osmotic Pressure Measurement

Eight milliliters of SWFI was added to a total of 200 mg of dermal filler sample, and osmotic pressure was measured using an osmometer (Fiske Associates 210, Advanced instruments, Norwood, MA, USA) after gently shaking and keeping the sample still for 10 min. Three independent samples from each dermal filler were analyzed.

### 2.6. Rheological Analysis

The rheological property of four dermal filler samples was measured using a rheometer (Anton Paar MCR 301, Graz, Austria). The measuring rotor was lowered until the gap between the upper and lower plates reached 2000 μm. A frequency of 5 Hz with a strain amplitude of 5 × 10^−4^ rad was conducted, and data were collected every 5 s. Measurements were performed at a shear rate of 1 s^−1^, with each shear rate tested 10 times to obtain an average value. Three independent samples from each dermal filler were measured.

### 2.7. Statistical Analysis

Differences in reconstitution test, osmotic pressure measurement, and rheological analysis were performed using one-way ANOVA (Analysis ToolPak add-in that is built in Microsoft Excel 2019) followed by Tukey’s post hoc tests for multiple comparisons. A *p* value of less than 0.05 was considered significant.

## 3. Results and Discussion

### 3.1. Functional Group Profiles of All Dermal Fillers Are Largely Similar

**Figure 1:**
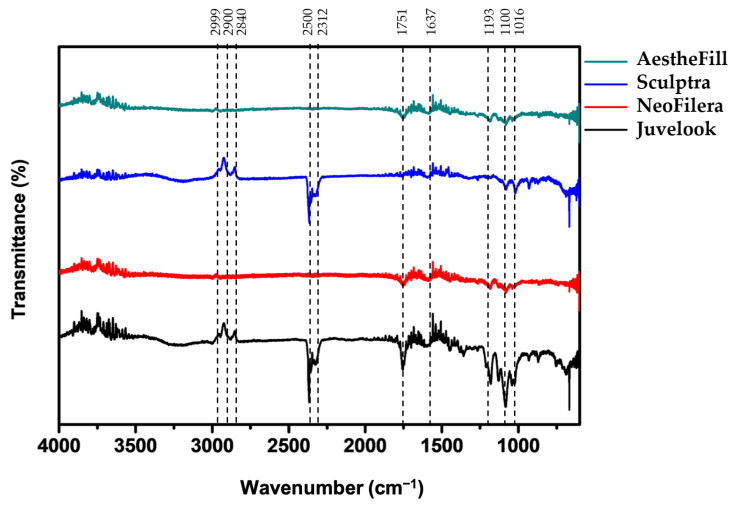
FTIR spectra of AestheFill (green line), Sculptra (blue line), NeoFilera (red line), and Juvelook (black line) dermal fillers.

All dermal fillers analyzed in this study contained PLA, and characteristic functional groups of carbon–hydrogen (C-H) and ester (COOH) were consistently observed at 2999 and 1751 cm^−1^, respectively, in the FTIR spectra (Figure 1). AestheFill, Sculptra, and NeoFilera also contain CMC, which exhibits hydroxyl (OH) at 3362, 1412, and 1316 cm^−1^; C-H alkanes at 2952 cm^−1^; carbonyl (C=O) at 1577 cm^−1^; and C-O carboxylic acid/ester absorbance bands at 1029 cm^−1^ [17]. However, only a broad peak between 1751 and 1637 cm^−1^ was distinctly observed among these expected bands (Figure 1).

In addition, three peaks in the 2840 and 2999 cm^−1^ range were present in Sculptra and Juvelook but not clearly visible in AestheFill or NeoFilera. A notable peak around 2500 cm^−1^ was also detected in both Sculptra and Juvelook (Figure 1), which was unusual for PLA-based samples. Juvelook is composed of PDLLA and HA, and previous studies have shown that OD (deuterium–hydrogen) or ND (deuterium–nitrogen) stretching vibrations can appear near this region in deuterated forms of hyaluronate [18]. However, the presence of this peak in Sculptra remains unclear.

Although all fillers exhibited a peak at 1751 cm^−1^, this peak was more intense in Juvelook. Both PLLA and PDLLA display a sharp absorbance at 1751 cm^−1^ attributed to carbonyl stretching [19]. Sodium hyaluronate shows an asymmetric stretching mode of planer carboxyl groups at 1616 cm^−1^, which shifts to 1735 cm^−1^ upon protonation [18], likely contributing to the sharper 1751 cm^−1^ peak observed in Juvelook. Multiple peaks between 1193 and 1016 cm^−1^ were observed in Juvelook, similar to AestheFill, Sculptra, and NeoFilera, although these peaks were more pronounced in Juvelook. Functional groups corresponding to C-O, C-C, and COH in hyaluronate have been reported in the 1200–950 cm^−1^ region [18], explaining the stronger signals in Juvelook compared with the C-O carboxylic acid/ester bands of CMC in the other fillers (Figure 1).

Overall, AestheFill and NeoFilera exhibited nearly identical FTIR spectra. Although Juvelook also contains PDLLA, distinct HA-related peaks such as OD or ND stretching vibrations at 2500 cm^−1^ and stronger C-C/COH bands between 1200 and 950 cm^−1^ were apparent. In contrast, the FTIR profile of Sculptra differed from those of AestheFill and NeoFilera but resembled that of Juvelook. The primary compositional difference was that Sculptra contains PLLA rather than PDLLA. Unlike nuclear magnetic resonance (NMR), which can distinguish stereoisomeric structures, FTIR detects only functional groups and cannot differentiate between PLLA and PDLLA. Therefore, the unique peak at 2500 cm^−1^ observed in Sculptra may result from the formation of new functional groups or shifts in existing group vibrations. The stereochemistry of these four PLA-based dermal fillers will require further investigation using NMR spectroscopy, chromatography, or mass spectrometry, because it has been shown that different stereochemistries of PLA result in different immune responses in macrophages, with variations in cytokine expression depending on their degradation products [20].

### 3.2. Microparticle Morphology Differs Markedly Among Dermal Fillers

**Figure 2:**
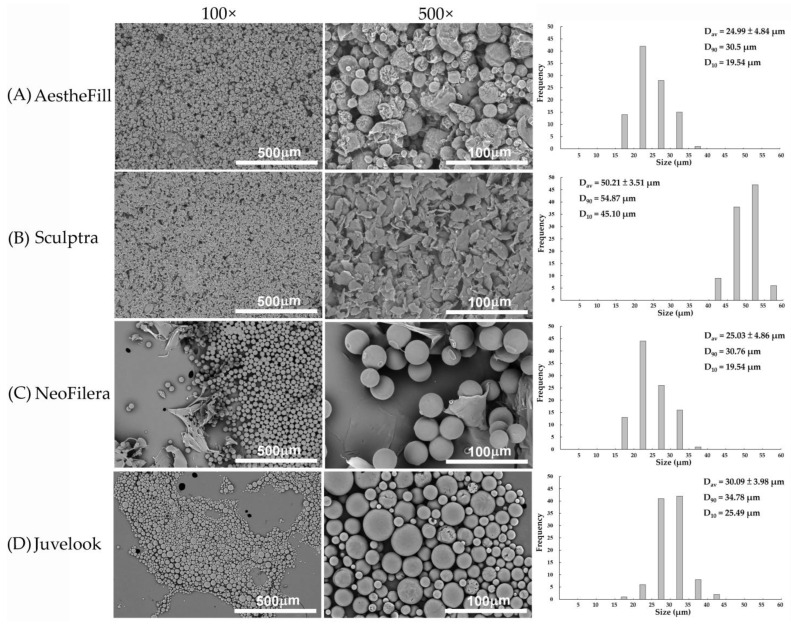
Representative scanning electronic microscope images of AestheFill (**A**), Sculptra (**B**), NeoFilera (**C**), and Juvelook (**D**) particles. Scale bar shows 50 μm with 100× magnification and 10 μm with 500× magnification.

The surface morphology of each dermal filler was examined using SEM, and the results are shown in Figure 2. Although both AestheFill and NeoFilera fillers are composed of PDLLA and CMC, their particle morphologies are distinct. AestheFill microparticles were predominantly spherical but exhibited wrinkled, rough surfaces with an average particle size of 24.98 ± 4.84 μm (Figure 2A). D_90_ in Figure 2A indicates that 90% of AestheFill particles had a size of 30.5 μm or less, and D_10_ means that 10% of AestheFill particles had a size of 19.54 μm or less. In contrast, the microparticles of NeoFilera and Juvelook displayed smooth surfaces (Figure 2C,D). NeoFilera microparticles had uniform sizes, although some flattened debris was observed (Figure 2C). The average particle size D_av_ of NeoFilera was 25.03 ± 4.86 μm, with D_90_ = 30.76 μm and D_10_ = 19.54 μm (Figure 2C). Juvelook particles showed greater size variability but maintained a consistently spherical morphology, with porous structures visible on the surfaces of some microspheres (Figure 2D). The average Juvelook particle size was slightly larger (30.09 ± 3.98 μm), with D_90_ = 34.78 μm and D_10_ = 25.49 μm. Previous studies have similarly reported multiple pores on Juvelook microspheres at magnifications of 2000× or higher [7,21]. Sculptra exhibited a distinct morphology compared with the other fillers; its particles were sheet-like in shape with rough surface features (Figure 2B). The average size of Sculptra particles was 50.21 ± 3.51 μm. Thus, despite having identical base materials, the morphology of AestheFill and NeoFilera differed substantially in surface characteristics but showed similarity in average particle size and size distribution. Additionally, NeoFilera and Juvelook displayed similar morphological features, including rounded shapes and relatively smooth surfaces, despite differences in their auxiliary components.

PDLLA is amorphous due to the random distribution of L- and D-lactic acid units in its polymer chain, while PLLA is semicrystalline, containing only L-lactic acid [21]. As a result, PDLLA is less brittle and exhibits better mechanical stability because its irregular structure prevents the formation of large crystalline domains. Consistent with these properties, only Sculptra showed a sheet-like morphology, whereas the three PDLLA-based fillers showed spherical morphology. The relatively larger and more irregular morphology of Sculptra particles was shown to contribute to prolonged tissue persistence and robust collagen stimulation [22]. These same features are associated with less homogeneous tissue dispersion and an increased risk of localized foreign-body reactions, including late-onset nodules when injected superficially or when inadequately reconstituted [22].

Although AestheFill, NeoFilera, and Juvelook all contain PDLLA, their surface morphology differs. AestheFill particles are rough, whereas NeoFilera and Juvelook microparticles are smoother. These differences may arise from variations in manufacturing processes rather than material composition alone. AestheFill is produced by suspending PDLLA in CMC followed by a solvent-spray process and subsequent lyophilization [23]. In contrast, a previous study demonstrated that particles generated by emulsification at a stirring speed of 200 rpm exhibited physicochemical characteristics similar to NeoFilera [8], suggesting that NeoFilera is likely manufactured via an emulsification process. The manufacturing method for Juvelook has not yet been published; however, its morphological similarities to NeoFilera imply that it may also be produced using an emulsification process, but this will require further investigation. Overall, the particle size and size distribution of the four dermal fillers predominantly ranged between 20 μm and 100 μm, a size range that has been reported to reduce the likelihood of macrophage phagocytosis and to favor fibroblast-mediated collagen neogenesis. Differences in particle morphology may further influence tissue response and handling characteristics. For example, the irregularly shaped particles of Sculptra have been associated with deeper injection planes and sustained bio-stimulatory effects in clinical practice. The rough-surfaced spherical particles of AestheFill may increase effective surface area, potentially enhancing cell interactions and collagen stimulation. In contrast, the relatively smooth-surfaced particles of NeoFilera and Juvelook may facilitate more uniform tissue integration and controlled collagen deposition. While these interpretations are supported by the prior literature and clinical observations, direct correlations between particle morphology and specific clinical outcomes require further investigation.

### 3.3. NeoFilera Exhibits the Shortest Reconstitution Time

**Figure 3:**
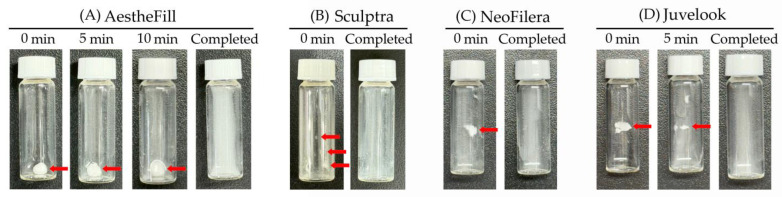
Pictures taken when SWFI is mixed with AestheFill (**A**), Sculptra (**B**), NeoFilera (**C**), or Juvelook (**D**) at 0 min, 5 min, 10 min, or when reconstitution is completed. Red arrows indicate when lyophilized or patty-like powders are not completely reconstituted.

All dermal fillers examined in this study were supplied either as large spherical lyophilized powders (red arrows in Figure 3A,C,D at 0 min) or as patty-like powders (red arrow in Figure 3B at 0 min). Before injection, these products must be reconstituted with sterile water for injection (SWFI) according to their respective manufacturer manuals. Therefore, the reconstitution time of each dermal filler was evaluated to better understand their clinical operability. When SWFI was added to a vial containing AestheFill lyophilized powder, the shape remained largely unchanged even after 10 min of standing (Figure 3A). The vial was then vortexed around 2 min until powder was evenly suspended in SWFI, resulting in a total time exceeding 15 min (Table 1). In contrast, the reconstitution times of Sculptra and NeoFilera were markedly shorter than that of AestheFill (Table 1); therefore, only the 0 min appearance was photographed (Figure 3B,C). Juvelook also demonstrated a relatively short reconstitution time of approximately 7.5 min (Table 1). At 5 min, the Juvelook powder mass had already decreased in size compared with 0 min (Figure 3D). Although Juvelook reconstituted significantly faster than AestheFill, it remained slower than Sculptra and NeoFilera (Table 1).

Reconstitution instructions differ slightly among products. Two hundred milligrams (mg) of AestheFill or NeoFilera powder are recommended to be suspended in either 1.4 or 8 mL of sterile water [24]. Sculptra required 367.5 mg of powder suspended in 5 mL of sterile water [25], whereas 50 mg of Juvelook powder is required to be suspended in 8 mL of sterile water according to manufacturer’s instructions [26]. To standardize comparisons, 25 mg of each powder was suspended in 1 mL of SWFI in this study, equivalent to reconstituting 200 mg of powder in 8 mL of SWFI. Consistent with previous studies [15], the reconstitution time of AestheFill powder was the longest. Reconstitution typically involves two steps: (1) hydration of PLLA or PDLLA powders and (2) dispersion of CMC (or HA) and PLLA (or PDLLA) microparticles following step 1 [16]. Although several modified techniques have been proposed to shorten the reconstitution time of AestheFill powder [15,16], these methods either require excessive physical effort from clinicians or increase the risk of contamination, leaving the problem unresolved. In contrast, NeoFilera, which consists of the same materials as AestheFill, displayed a dramatically shorter reconstitution time. This difference is likely attributable to manufacturing processes. AestheFill particles are produced by suspending PDLLA in CMC via a solvent-spray technique, resulting in a CMC coating primarily on particle surfaces. The hydrophobic PDLLA core may therefore hydrate slowly even though CMC dissolves readily. Conversely, the emulsification process likely used for NeoFilera enables more thorough intermingling of PDLLA and CMC, facilitating rapid hydration and dispersion.

Sculptra contains mannitol in addition to CMC, and previous reports suggest that mannitol helps prevent CMC clumping and improves suspension homogeneity [27]. However, other studies recommend prolonged hydration to avoid formation of large CMC micro-lumps [28]. In this study, reconstituion time was recorded based on visual observation of complete dispersion; whether micro-lumps persisted should be further examined via optical microscopy. Although Juvelook required more than 5 min to reconstitute, its performance was nevertheless much faster than AestheFill, suggesting that Juvelook PDLLA readily hydrates and disperses into HA solution. Reconstitution time may also be influenced by the altered powder-to-water ratio used in this study. For instance, the manufacturer recommends adding 1 mL SWFI per 73.5 mg of Sculptra powder, but only 25 mg was used here. This modification demonstrated that fewer PLLA particles were required for hydration and dispersion, likely accelerating reconstitution. Conversely, Juvelook was intended to use 1 mL SWFI per 7 mg of powder, but 25 mg was used here, indicating that reconstitution may be even faster under the manufacturer’s recommended ratio. To better reflect real clinical practice, future experiments should follow each product’s original powder-to-water ratio during reconstitution. However, reconstitution time in this study was determined by visual observation, which is inherently subjective and not fully quantitative, potentially introducing observer-dependent variability. More objective and quantitative approaches, such as microscopic analysis, ultraviolet-visible spectrophotometric measurement of turbidity, or tracking of fluorescently labeled microparticles, should be employed in further studies to enable more precise and reproducible comparisons. For clinical application, faster and more complete hydration can decrease the likelihood of undissolved microaggregates, which are associated with uneven injection and palpable nodules. However, injectability and clinical performance may be altered if off-label dilutions occur, suggesting that manufactuers need to take reconstitution performance of PLA-based dermal fillers into account.

### 3.4. Viscosity Reveals Distinct Physicochemical Characteristics Among Dermal Fillers

**Figure 4:**
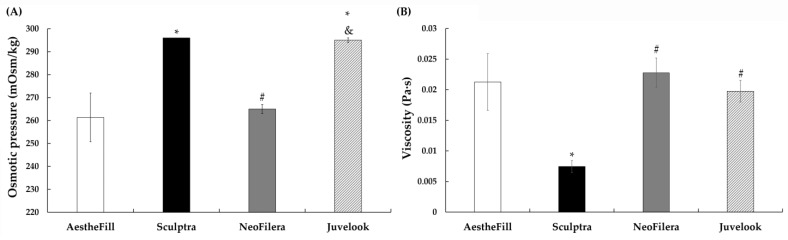
The result of osmotic pressure (**A**) or viscosity (**B**) for AestheFill (white bars), Sculptra (black bars), NeoFilera (gray bars), or Juvelook (slashed bars) dermal fillers. In (**A**), * *p* = 0.030 or 0.031 when compared osmotic pressure of Sculptra or Juvelook against AestheFill; ^#^ *p* = 0.001 when compared osmotic pressure of NeoFilera against Sculptra; and ^&^ *p* = 0.002 when compared osmotic pressure of Juvelook against NeoFilera. In (**B**), * *p* = 0.031 when viscosity of Sculptra is compared against AestheFill; ^#^ *p* = 0.003 or 0.001 when viscosity of NeoFilera or Juvelook is compared against Sculptra.

In order to avoid hyperosmotic or hypotonic reactions after injection, the osmotic pressure of the four dermal fillers was examined. The osmotic pressure of AestheFill was 261.33 ± 10.60 mOsm/kg, while NeoFilera was 265.00 ± 2.00 mOsm/kg, similar to AestheFill (Figure 4A). In contrast, Sculptra and Juvelook exhibited significantly higher osmotic pressures of 296.00 ± 0.00 and 295.00 ± 1.00 mOsm/kg, respectively. Because normal serum osmotic pressure ranges from 275 to 295 mOsm/kg [29], AestheFill and NeoFilera each became a hypotonic solution after reconstitution. Injection of hypotonic solution may induce discomfort and swelling at the injection site [25]. Although reconstitution with normal saline could raise osmotic pressure, saline has been reported to cause CMC micro-lumps and incomplete reconstitution [27]. Therefore, warnings regarding potential discomfort and swelling should be included in the instruction sheets for AestheFill and NeoFilera. In contrast, Sculptra and Juvelook showed osmotic pressures within the physiologic range, suggesting minimal irritation upon injection.

The viscosities of AestheFill, NeoFilera, and Juvelook were comparible, with values of 0.021 ± 0.004, 0.023 ± 0.002, and 0.020 ± 0.002 Pa·s, respectively (Figure 4B). Sculptra displayed a substantially lower viscosity of 0.007 ± 0.001 Pa·s, likely due to the higher dilution ratio of SWFI to PLLA particles. A shear rate of 1 s^−1^ was used because these materials are particulate suspensions rather than pure liquids; PLLA or PDLLA particles dispersed in CMC or HA cannot maintain a stable flow at high shear rates. Unlike HA fillers, the viscosity profiles of PLLA/PDLLA fillers have rarely been reported. Knowledge of HA filler viscosity helps clinicians choose suitable fillers based on clinical needs. For instance, high-viscosity HA fillers are injected into deep fat compartments to provide stable volume without affecting muscle movement [12]. In this study, all PLLA/PDLLA fillers behaved as low-viscosity fluids, suggesting that they can be injected easily. Clinically, the characteristics of low viscosity align with these fillers’ established use as a collagen bio-stimulator rather than a contour-defining filler. However, osmotic pressure and viscosity were measured under simplified in vitro conditions that do not fully replicate the dynamic in vivo environment after injection; thus, collecting more case reports or investigations of clinical performance should be further required.

AestheFill dermal filler was approved by the Food and Drug Administration (FDA) in the United States in 1984, followed by Sculptra dermal filler in 2004 [1]. Mild adverse reactions such as redness, nodules, bruising, granulomas, or papules have been reported from injecting AestheFill or Sculptra dermal fillers, but no life-threatening events have been reported, supporting the clinical safety of both products [3,30,31,32,33,34]. Juvelook dermal filler received CE marking in 2020 in Europe [35], and reported adverse events such as noninflammatory nodules, mild swelling, redness, and petechiae were self-resolving [7,10]. NeoFilera dermal fillers were launched in Thailand in 2024 and approved in Taiwan in 2025 [8], with no clinical case reports or complications documented to date. Although all four fillers share a common PLA-based bio-stimulatory mechanism and their clinical safety is established, their distinct physicochemical profiles suggest that they are not interchangeable in practice. Differences in composition, particle morphology, reconstitution time, osmolality, and viscosity collectively influence ease of use, patient comfort, tissue integration, and possibly long-term outcomes. The present findings provide a mechanistic basis for tailoring product selection to specific clinical indications, injection planes, and patient sensitivities, thereby supporting more personalized and predictable aesthetic treatments.

## 4. Conclusions

This study systematically investigated the physicochemical characteristics of four current PLA-based dermal fillers, including three PDLLA-based products (AestheFill, NeoFilera, and Juvelook) and one PLLA-based filler (Sculptra). NeoFilera and AestheFill exhibited comparable functional groups, averaged particle size, size distribution, osmotic pressure, and viscosity but differed markedly in reconstitution time, likely due to differences in PDLLA particle–CMC integration during manufacturing. Juvelook demonstrated unique PDLLA particle morphology and a moderate reconstitution time, whereas Sculptra displayed the most distinct physicochemical profile, including larger particle size, low viscosity, and high osmotic pressure, which may be attributable to its PLLA-based formulation and inclusion of mannitol. Although the relationship between physicochemical characteristics and clinical outcomes requires further investigation, the observed differences among PLA-based dermal fillers are likely to influence their handling properties, injectability, tissue integration, and early post-injection behavior. In particular, variations in viscosity, particle morphology, and size may affect injection force and collagen stimulation patterns. Differences in reconstitution time and osmotic pressure may affect preparation efficiency and immediate tissue response. This comparative physicochemical analysis provides clinically relevant insights that may assist clinicians in selecting PLA-based fillers adapted to specific treatment goals and patient needs, thereby supporting more predictable and optimized aesthetic outcomes.

## Figures and Tables

**Table 1:** Reconstitution time for AestheFill, Sculptra, NeoFilera, and Juvelook is demonstrated as average ± standard deviation. * *p* indicates statistically significance.

Dermal Fillers	AestheFill	Sculptra	NeoFilera	Juvelook
Reconstitution (seconds)	966.3 ± 37.6	250.0 ± 41.3	140.7 ± 32.9	450.7 ± 36.7 *
*p* value when comparing against reconstitution time of AestheFill	N.A.	2.6 × 10^−5^ *	1.0 × 10^−5^ *	7.0 × 10^−5^ *
*p* value when comparing against reconstitution time of Sculptra	N.A.	N.A.	0.025	0.0034 *
*p* value when comparing against reconstitution time of NeoFilera	N.A.	N.A.	N.A.	0.0004 *

## Data Availability

Data are contained within the article.

## Abbreviations

The following abbreviations are used in this manuscript:

PLA	Polylactic acid
PDLLA	Poly(D,L-lactic acid)
PLLA	Poly(L-lactic acid)
CMC	Carboxymethylcellulose
HA	Hyaluronic acid
FTIR	Fourier-transform infrared spectroscope
SEM	Scanning electron microscope
SWFI	Sterile water for injection
